# Healthcare providers' experiences screening for intimate partner violence among migrant and seasonal farmworking women: A phenomenological study

**DOI:** 10.1111/hex.12421

**Published:** 2015-11-04

**Authors:** Jonathan B. Wilson, Damon L. Rappleyea, Jennifer L. Hodgson, Andrew S. Brimhall, Tana L. Hall, Alyssa P. Thompson

**Affiliations:** ^1^ Oklahoma Baptist UniversityShawneeOKUSA; ^2^ East Carolina UniversityGreenvilleNCUSA; ^3^ University of OklahomaNormanOKUSA

**Keywords:** intimate partner violence, migrant farmworker, seasonal farmworker, screening, health care

## Abstract

**Background:**

Migrant and seasonal farmworking (MSFW) women patients experience substantially more intimate partner violence (IPV) than the general population, but few health‐care providers screen patients for IPV. While researchers have examined screening practices in health‐care settings, none have exclusively focused on MSFW women.

**Objective:**

The aim of this phenomenological study was to explore the experiences of health‐care providers who have screened for and/or addressed IPV with MSFW women patients.

**Design:**

Researchers utilized descriptive phenomenology to capture the lived experiences of these health‐care providers. Data were analysed using Colaizzi's seven‐stage framework.

**Setting and participants:**

Interviews were conducted with nine female participants – all of whom: (i) were clinically active health‐care providers within the MSFW community, (ii) were bilingual in English and Spanish or had access to a translator, (iii) had treated MSFW patients who had experienced IPV and (iv) were at least 18 years of age.

**Results:**

Participants' experiences were reflected in four emergent themes: (i) provider‐centered factors, (ii) patient‐centered factors, (iii) clinic‐centered factors and (iv) community‐centered factors. Participants described barriers to establish routine IPV assessment, decrease patient ambivalence and increase on‐site support and community resources.

**Discussion and conclusions:**

This study aimed to generate a greater understanding of the experiences of health‐care providers with screening for and addressing IPV with MSFW patients. Implications and recommendations for research, clinical practice and policy are provided.

## Introduction

Intimate partner violence (IPV) is a pervasive public health problem^1^ with serious consequences for women's health.^2^ Migrant and seasonal farmworking (MSFW) women are particularly at risk of experiencing IPV because of cultural beliefs, environmental factors, and health disparities.^3^, ^4^, ^5^ Despite previous researchers indicating that IPV screenings in healthcare settings increase IPV identification rates,^6^, ^7^ and previous reports that MSFW women report higher rates of IPV than the general population,^8^ many providers opt not to screen for various reasons.^9^, ^10^ The authors examined providers' experiences screening MSFW women for IPV in healthcare settings.

## Literature review

IPV is considered to be the primary cause of injury to all women ages 15–44^11^ and has resulted in significant inpatient and outpatient health costs (e.g., medical costs incurred from IPV injuries) and devastating social and family intergenerational consequences.^12^ Among MSFW women, physical violence victimization ranged from 18.5^8^ to 20%^13^ within the previous year and between 19^14^ and 33.9%^8^ over one's lifetime. Hazen and Soriano^8^ also identified a 20.9% lifetime prevalence rate of sexual coercion among MSFW women, with 14.4% reporting having experienced it within the previous year alone.

### Risk factors for IPV

MSFW are among some of the most disadvantaged and medically underserved populations in the United States.^15^ Various factors including poverty, frequent mobility, low literacy, language and cultural barriers impede MSFW access to social services and cost‐effective primary health care.^16^ Awareness of available resources (e.g., women's shelters, police, domestic violence hotlines) among MSFW women appears to be low, with only 22% of being aware of resources, yet 87% indicating they would seek help if available.^17^ Other variables of work stress (i.e., low income, unemployment)^18^ and male work status (i.e., ‘low‐status’ jobs)^17^ have been associated with IPV as well.

### IPV screening

Routine screening for IPV in health‐care settings could identify women at risk and lead to interventions that reduce violence and improve health outcomes.^6^ Although IPV screening is recommended by the Institute of Medicine^19^ and several professional organizations (e.g., American Congress of Obstetricians and Gynecologists^20^), most providers do not routinely screen for IPV.^21^ Providers reported numerous barriers to screening for IPV including training,^21^, ^22^ lack of time and referral resources,^21^ self‐assessed competence in identifying IPV^9^ and lack of confidence in the ability to make referrals, discomfort in asking the IPV screening questions and no ready access to mental health specialists.^9^ Additional barriers to disclosing IPV among MSFW patients included feeling ashamed or embarrassed,^23^ inability to speak English and no access to a translator,^23^ fear of being deported or separated from family,^14^, ^24^ fear that a perpetrator would find out and make things worse^23^ and illiteracy.^25^ Given that much of the available data is at least a decade old, it is not known how providers are experiencing screening for IPV and why some continue to screen in the face of seemingly insurmountable barriers. Furthermore, while researchers have recently examined screening practices in health‐care settings, determining that many providers do not screen for IPV^6^, ^21^, ^26^; none have exclusively focused on MSFW women, a highly at‐risk population for IPV.^8^ The purpose of this study was to examine health‐care providers' experiences screening for and treating IPV among MSFW women patients.

## Method

Data analysis was conducted using Colaizzi's^27^ seven‐stage phenomenological analysis framework. A descriptive phenomenology^28^ approach was chosen to allow in‐depth exploration and examination of the experiences of health‐care providers' experiences screening for IPV among MSFW patients. The use of one primary open‐ended question was utilized for the purposes of this study: What is the essence of health‐care providers' experiences when screening for and treating IPV in the MSFW women population? Phenomenology is about searching for meanings and essences of experiences using first‐person accounts during in‐depth informational one‐to‐one interviews, transcribed and analyzed for meanings and themes.^27^, ^29^, ^30^ By capturing and describing the lived experiences of healthcare providers with experience screening for IPV among MSFW patients, researchers sought to fill a gap in the literature on this vulnerable and underserved population where there is limited knowledge. The goal was to first document the experiences of providers are able to screen for IPV despite all the challenges for this population present in the health‐care system.

### Sample

Purposive sampling techniques were used to recruit participants from across the United States. The Migrant Clinicians Network (MCN; www.migrantclinician.org), an organization that serves health‐care providers who serve MSFW patients and their families, assisted the primary investigator (PI) with recruiting participants from within their network of members via an email listserv. The MCN assisted in distributing recruitment information nationally so a broader sample could be obtained from across the migratory stream, increasing efforts towards thematic saturation. According to Macnee,^31^ thematic saturation occurs when the analysis of additional data (in this case, participant interviews) yields no new themes. To be enrolled in the study, participants had to: (i) be clinically active healthcare providers who serve the MSFW community, (ii) be bilingual in English and Spanish or have access to a translator, (iii) have treated MSFW patients who have experienced IPV and (iv) be 18 years of age or older. Saturation was achieved for this study at nine participants. All nine participants were female health‐care providers ranging in age from 29 to 75 years. Three participants were bilingual in English and Spanish, and six participants spoke English only. Six participants were White, two were Hispanic/Latino, and one was African American. Participants were from three different regions in the United States.

### Data collection and analysis

Due to the national reach of sample participants, data were collected via phone or Skype interviews. The PI employed an interview guide (Table 1) to structure the interview. The interview guide method^32^ lists questions to be explored and is designed to ensure that each interview follows the same basic format. The probing questions were based on findings from a policy brief examining current IPV research on the MSFW population^33^ and on the principles of biopsychosocial–spiritual model.^34^, ^35^, ^36^ A model used to advocate that health is a biomedical, psychological, social and spiritual dimension.

**Table 1:** Interview guide

Category	Question
Grand tour question	How would you describe your experience caring for migrant and/or seasonal farmworking women patients who have experienced intimate partner violence?
Probing questions	In your experience, how prevalent is intimate partner violence among this population?
	At what point during the visit is intimate partner violence typically addressed? Who usually brings up the topic of intimate partner violence (the provider or the patient)?
	What screening methods do you use to detect intimate partner violence and how do you introduce them to your migrant and/or seasonal farmworking patient population?
	What protocols do you follow for determining who and when to screen?
	How comfortable do you feel with recognizing and effectively responding to intimate partner violence? Is there anything that might increase your comfortability in this matter?
	What has been the most challenging in your experiences screening for and/or addressing intimate partner violence with migrant and/or seasonal farmworking women?
	In your opinion, what are the ethical implications of asking about intimate partner violence?
	Are there any special considerations you keep in mind when working with migrant and/or seasonal farmworking women compared to other cultural groups? If so, what are they?
	Is there anything else that you would like to share about these experiences? If so, what?

Data analysis was carried out using Colaizzi's^27^ seven‐stage phenomenological analysis framework. To become familiar with the data, the PI listened to each audio recording and read each transcript several times. Significant statements were then extracted from the transcripts directly, and each statement was assigned a formulated meaning. Common formulated meanings became evident and were organized into thematic clusters. Investigators were able to achieve 100% agreement on the findings at the conclusion of the analysis process. When there was a disagreement (13 times), each investigator would share his or her perspective, both would re‐examine the raw data, and one or the other investigator would adjust his/her interpretation until both parties were able to reach a satisfactory agreement.

### Verification processes

When conducting qualitative research, it is imperative that the investigator employs strategies for verifying the data's trustworthiness.^37^ Therefore, all investigators prepared bias statements and referenced them regularly throughout the analysis process. For example, the PI identified biases related to being a White, heterosexual, upper‐middle class male who has never personally experienced IPV. In addition, a triangulated researcher^37^; (a second researcher who coded independently from the PI); reflexive journal^37^ (used to bracket each investigator's experiences throughout the study) and audit trail^37^ (used to note emerging themes and analysis processes) were also utilized to reduce the possibility that biases or deviations from Colaizzi's^27^ framework could unintentionally influence or alter the data analysis process.

## Results

The results revealed 391 significant statements, 108 formulated meaning statements, 13 thematic clusters and four emergent themes, which reflect the essence of the experiences of screening for IPV among MSFW women patients for health‐care providers. The emergent themes revealed by this study include the following: (i) provider‐centered factors, (ii) patient‐centered factors, (iii) clinic‐centered factors and (iv) community‐centered factors. Under each emergent theme below, a brief overall summary statement and detailed summary of each thematic cluster is provided. A summary of the thematic clusters and emerging themes is provided in Table 2. Table 3 illustrates examples of Colaizzi's stages^27^ in action, with four significant statements and their associated formulated meanings, thematic clusters and emergent themes. Lastly, an exhaustive description was developed from the findings to highlight the essence of the participants' lived experiences and reflect the essential structure of the phenomena under investigation and is displayed in Table 4.^27^

**Table 2:** Emergent themes and thematic clusters

Emergent themes	Thematic clusters
Provider‐centered factors	Health‐care providers use various IPV screening protocols with MSFW patients
	Health‐care providers respond to patient disclosures of IPV in various methods
	Health‐care providers experience barriers to screening for and addressing IPV with MSFW patients
	Health‐care providers believe change is needed to improve MSFW patient care
	Health‐care providers are confronted with the partners of their patients
Patient‐centered factors	MSFW patients experience IPV in numerous forms
	MSFW patients respond to IPV perpetration in various ways
	MSFW patients experience barriers to disclosing IPV and seeking resources suggested by providers
Clinic‐centered factors	Some clinics have protocol/resources in place to address IPV with patients
	Some clinics unintentionally create barriers to effectively address IPV with patients
Community‐centered factors	IPV in the MSFW community is a multifaceted problem
	Unique cultural factors within the MSFW community may exacerbate IPV
	Communities provide resources to aid MSFW women experiencing IPV
	Outcomes for IPV victims and perpetrators vary within the MSFW community

**Table 3:** Selected examples of narratives and emergent theme formation

Significant statements	Formulated meanings	Thematic clusters	Emergent themes
‘I would definitely address it if the answer was yes or if the patient brought it up to me. If I suspected it I would address it, but I wouldn't go fishing for it…’	Provider specifies the time of and/or frequency of IPV screening	Health‐care providers use various IPV screening protocols with MSFW patients	Provider‐centered factors
‘I don't have any facts but… a lot of our patients are undocumented, so calling the police and sending their spouse to jail where there's the possible deportation or on the other side where [our patients] might get deported. That's a huge thing for people’	Immigration status of patients (including fear of deportation) is a barrier for patient disclosures of IPV	MSFW patients experience barriers to disclosing IPV and seeking resources suggested by health‐care providers	Patient‐centered factors
‘But I think when I first came here I did bring it up… and then I kind of backed off because I thought they'd think I'm crazy. Like, “Look at all the things we could be doing”’	Provider experienced resistance from employer regarding IPV screening/response protocol	Some clinics unintentionally create barriers to effectively addressing IPV with patients	Clinic‐centered factors
‘I hear other patients talking about how their husbands expect them to have food on the table and expect them to do this or that or the other with the children, which I don't hear my non‐migrant patients talking about…’	Traditional gender roles among the MSFW population (i.e. machismo) exacerbate IPV	Unique cultural factors within the MSFW community may exacerbate IPV	Community‐centered factors

**Table 4:** Exhaustive description

Health‐care providers who serve the MSFW community display considerable passion, dedication and commitment towards caring for MSFW women who have been victimized by IPV. Despite their desire and willingness to lend aid, many health‐care providers feel unequipped (e.g., lack of IPV training, lack of awareness of available IPV resources) to respond in such a manner that equips their patients with the knowledge and resources necessary to escape dangerous relations, and face several barriers to screening for and addressing IPV with MSFW patients, some of which are provider‐related (e.g., inability to speak Spanish), some patient‐related (e.g., lack of patient accessibility) and some clinic‐related (e.g., lack of required IPV screening protocol)
Although many health‐care providers feel confident in their abilities to discuss IPV with MSFW patients, most indicated a sense of uncertainty in their ability to truly help their patients without placing them at risk for further abuse. Because MSFW patients often present for their medical visits with their partners, health‐care providers struggle to effectively and discreetly screen for and address IPV with their patients. Providers believe that being as educated and informed as possible about the multifaceted problem of IPV among the MSFW community is essential. Provider trainings are one method in which to better educate health‐care providers about IPV among the MSFW population
Health‐care providers recognize the complexities and pervasiveness of IPV among the MSFW community. Not only does IPV take multiple forms among MSFW patients (e.g., physical violence, rape, abuse during pregnancy, abuse by non‐partner), but variability is evident in the ways that MSFW women respond to IPV as well. Additionally, just as health‐care providers experience barriers to screening for and addressing IPV with MSFW patients, providers observe numerous barriers to disclosing and responding to IPV faced by MSFW patients
Health‐care providers also encounter clinic and community‐centered factors that influence their abilities to effectively screen for and address IPV among their MSFW patients. Despite the common perception among participants that IPV among the MSFW community is much more prevalent than the general population, and the many unique cultural factors among MSFW families that exacerbate IPV (e.g., traditional gender roles), variability is evident in the amount of support providers receive from the communities and health‐care clinics in which they serve. These health‐care providers consider a multidisciplinary team approach to be an important element in the management of MSFW patients who have experienced IPV

### Emergent theme 1: Provider‐centered factors

Participants shared their personal experiences with implementing and utilizing IPV screening protocols, responding to patient disclosures of IPV and encountering barriers to screening for and addressing IPV with their patients. The following thematic clusters illustrate these experiences.

#### Thematic cluster 1a: Screening protocols

Participants discussed various components of the IPV screening process, such as determining when, whom, how (e.g., verbal or written, how frequently) and where to screen. Eight participants indicated that they typically administer verbal IPV screenings. Brenda described one of the screening questions she usually administers, ‘Have you ever been hit, kicked, slapped called names? … it's very, very specific’. Some participants indicated that they would screen for IPV if they suspected it. For example, Erin indicated, ‘I would definitely address it… if the patient brought it up to me. If I suspected it I would address it, but I wouldn't go fishing for it’. Finally, Connie described her experience discovering IPV while the patient was in labour/delivering a child:

I realized I had to ask her to move her hair…she had this long thick hair that she had wrapped all around her. And I had to lure her boyfriend out of the room… and anyways … here she is 9 months pregnant having a baby and he had tried to kill her. He had tried to strangulate her. She had these horrible bruise marks all over her neck and her chest. Oh my god it was horrible.

#### Thematic cluster 1b: Provider response to patient disclosures of IPV

Participants described several aspects of their own experiences responding to MSFW patient disclosures of IPV. All nine participants commented on their self‐assessed confidence/comfort with screening for and responding to IPV with their MSFW patients. Lucy commented, ‘I have no trouble having the conversation about what's going on with them. Now, I do not feel perfectly comfortable figuring out what to do about it’. Four participants described their experiences encouraging patients to advocate for themselves. Donna shared, ‘I can make clear that that is not acceptable “that someone pounds you because you didn't cook the right frijoles”’. Three participants described their experiences with formulating a safety plan with patients. Erin discussed the challenge of creating a safety plan with patients you only see once or twice, ‘Something that I try to do is… develop a plan… often times especially with migrant and seasonal workers you're only seeing them one time… or maybe twice, and then you're not sure where they're going to be going next’.

#### Thematic cluster 1c: Provider‐reported barriers to IPV screening

All participants indicated barriers that make IPV screening among MSFW patients more difficult. Five participants reported lack of resources (or awareness of resources) available to patients as a barrier to screening for IPV. For example, Lucy described her experience, ‘When it comes time to figure out, “Okay well what are we going to do about this?” That's where I feel like I'm not equipped… I feel equipped to talk but not equipped to act’. Three participants described their inability to speak Spanish as a barrier to IPV screening. Additionally, three participants identified patient accessibility as a barrier to screening for and addressing IPV. Karen described her experience, ‘I'm not able to follow‐up and I don't know what's going on, if they're okay, or if they need further assistance. I can't keep in contact with them because they're migrant.

#### Thematic cluster 1d: Access to resources/services

Participants commented how access to various resources for MSFW patients experiencing IPV both on‐site and out in the community can be helpful. Two participants believed that access to additional professionals (e.g., social worker, medical family therapist) could be helpful in addressing IPV with patients. Two participants described their experiences with providing patients contact information for local resources. Donna described a resource developed by her colleagues for MSFW patients who have experienced IPV, ‘We also developed…a tiny handout that can't be more than 2 by 4 [inches]… that they could tuck in a bra… that gave the phone number for the domestic violence [hotline]’.

#### Thematic cluster 1e: Health‐care system improvements for MSFW IPV treatment

Participants discussed changes that they believed necessary, ranging from health‐care provider trainings to adaptable, culturally sensitive IPV response protocols. Erin indicated, ‘I just wish I had like more…knowledge about it or better ways to go about it… I feel like I haven't had any real training on it so I'm just doing [my] best’. Bonnie also suggested the utility of provider trainings about IPV among MSFW patients, ‘[My employer] could offer something… Maybe once a year, if… [my employer] wanted to bring in someone to bring up the latest resources and the latest things that are available’.

#### Thematic cluster 1f: Providers are confronted by patients' partners

Participants discussed their experiences interacting with MSFW patients' partners and the impact that these interactions have had on their attempts to address IPV with patients. Two participants described attempting to separate partners from patients. Karen described, ‘Somehow we figured out a way to keep the husband in the waiting room and got the patient back to the exam room by herself’.

### Emergent theme 2: Patient‐centered factors

Participants shared several patient‐centered factors (i.e., lived experiences and circumstances of MSFW patients observed by participants) pertaining to IPV screening and treatment of their MSFW patients including various forms of IPV, differences in responses to IPV perpetration and barriers to IPV disclosure among MSFW women. The following thematic clusters illustrate these factors.

#### Thematic cluster 2a: Various forms of IPV among MSFW patients

Participants described their experiences treating multiple forms of IPV presented by MSFW patients. Three participants indicated that IPV often occurs during pregnancy within the MSFW community. Brenda commented, ‘They often start… actually the abuse during pregnancy… it's a big deal’. Connie added that violence during pregnancy often occurs by men other than the patient's partner as well. Participants indicated that violence does not necessarily stop after a patient discloses IPV either. For instance, Lucy described one experience where a patient had reported her partner for IPV and he was subsequently deported. Later, the patient's partner was threatening her family of origin with violence, and the partner's family was threatening the patient with violence as well.

#### Thematic cluster 2b: Responses to IPV among MSFW women

Participants described their experiences observing the responses of MSFW women to being victimized by IPV by their partners and being screened for IPV by their health‐care providers. Connie added, ‘I've had…over the years maybe 10 or 15 women who've… admitted to what was going on but…couldn't do anything about and didn't want to do anything about it and weren't willing to accept any kind of help’. Carol indicated that many patients blame themselves for the violence they are enduring. Similarly, Connie explained that some patients simply refuse to accept services or assistance from their health‐care providers.

#### Thematic cluster 2c: Barriers to disclosing IPV or seeking resources

Perhaps one of the most heavily discussed thematic clusters involved barriers for MSFW patients to disclosing IPV and seeking resources to help end the violence. Eight participants indicated that having partners or other family members in the room is often a barrier to IPV disclosure for patients. Sharon spoke of her experience, ‘I noticed that if the husbands come in with them, [the patients] don't say anything’. Bonnie added, ‘A lot of times the partner is present, because he's the one paying… So, being careful because even you're asking him to step out it may already send red flags to him’. Seven participants indicated immigration status of patients (including fear of deportation) as a barrier to disclosing IPV. Erin described her experience, ‘A lot of our patients are undocumented, so calling the police and sending their spouse to jail where there's the possible deportation or on the other side where [our patients] might get deported. That's a huge thing for people’. Other barriers reported by participants include lack of transportation, language barriers, confidentiality/privacy concerns and gender of health‐care provider.

### Emergent theme 3: Clinic‐centered factors

Participants revealed clinic‐centered factors including screening protocol/resources in place to address IPV with patients and unintentionally created barriers to effectively addressing IPV with patients created by some clinics. The following thematic clusters illustrate these factors.

#### Thematic cluster 3a: Clinic protocol/resources for IPV screening

Participants described available resources within their clinics to assist providers in addressing IPV with MSFW patients. Six participants indicated that they refer patients to on‐site social workers, medical family therapists, etc. if IPV is disclosed or if they feel uncomfortable addressing IPV with their patients. For example, Brenda mentioned a few such resources available on site in her clinic, ‘We have a whole behavioural health component of our clinic so they can get free counselling services… We have our own social worker that specializes… in prenatal and perinatal issues’. Three participants indicated that interpreters had enabled them to administer IPV screenings to MSFW patients who do not speak English.

#### Thematic cluster 3b: Clinics can create barriers

While most participants indicated that the clinics at which they work have some resources available to assist with screening for and addressing IPV with their MSFW patients, some participants indicated that the clinics could uninten‐tionally create barriers for providers as well. Carol shared that she was once turned away by her employer when she suggested a change in the way that her clinic currently addressed IPV, ‘I think when I first came here I did bring it up… and then I kind of backed off because I thought they'd think I'm crazy. Like, “Look at all the things we could be doing”’.

### Emergent theme 4: Community‐centered factors

Participants discussed the unique community‐centered factors associated with IPV within the MSFW community including IPV prevalence, available resources for MSFW women experiencing IPV, and outcomes for IPV victims and perpetrators within the MSFW community. The following thematic clusters illustrate these factors.

#### Thematic cluster 4a: IPV is a considerable problem

Participants discussed the prevalence of IPV among the MSFW community, and all nine participants agreed that the prevalence of IPV among the MSFW community is substantial. Sharon indicated, ‘I think [IPV] is pretty common with the migrant [population]’. Carol added, ‘I mean I hate to say a lot but we have [women who have experienced IPV] frequently’. Brenda concurred, ‘I am afraid that [IPV among this population] is very high’.

#### Thematic cluster 4b: MSFW cultural factors exacerbate IPV

Participants discussed numerous unique cultural factors in the MSFW community that exacerbate IPV. Seven participants described how traditional gender roles in the MSFW community influence the prevalence of IPV among their patients. Three participants described that the presence of children in the family increased their overall sensitivity and desire to screen for IPV. Karen specified:

Of course I'm considering if…there's domestic violence going on with the partner, if there is going to be domestic violence going on with the children as well… That makes it…an easier end road for reporting and for getting the process started because if there is [domestic violence] … it's not necessarily a reportable offense for an adult but it is a reportable offense for a child.

Bonnie described that patients within the MSFW community normalize IPV as part of life and that she often worries that violence will extend beyond the partner relationship to other members of the family. Brenda emphasized that the MSFW community maintains a cultural independence from mainstream society. Additionally, Donna explained that stressors associated with immigration status and occupational stressors among IPV perpetrators may exacerbate IPV.

#### Thematic cluster 4c: Communities provide resources to aid IPV victims

Participants discussed various resources available within their communities for MSFW patients who have experienced IPV. Three participants indicated having access to local resources within the community to aid MSFW women experiencing IPV. One participant, Donna, later described one case example where she actually drove one of her patients from a dangerous home environment to a local shelter, ‘We had the capacity to just load up that family and bring them back to [my town] about 150 miles from the small city, which we did’.

#### Thematic cluster 4d: Outcomes for IPV perpetrators vary

Participants described a few different outcomes that they had observed for IPV perpetrators in their experiences. Four participants indicated that IPV perpetrators were required to serve jail time as a result of their violence. One participant, Lucy, reported that the partner of one of her patients was actually deported as a result of his violent behaviours, ‘She reported her husband, and he was deported for the domestic violence’.

## Discussion

This study aimed to generate a greater understanding of health‐care providers' experiences screening for and addressing IPV with MSFW patients. Given that MSFW patients experience greater levels of IPV than the general population,^8^ and the fact that no known studies have been published regarding IPV screenings of MSFW women in health‐care settings, this study fills an important gap in the literature. This study illustrates the unique challenges experienced by health‐care providers of MSFW patients who have experienced IPV. Although most participants in this study expressed their sincere desire to lend aid to their patients victimized by IPV, many felt uninformed and unequipped to begin to broach a problem so culturally engrained.

### Provider‐centered factors

While some participants utilized brief written or verbal screening tools for IPV (some with only one question), other participants engaged in open discussions with their patients about IPV. Consistent with previous research, participants reported several barriers to screening for IPV including training,^22^, ^27^ lack of time and referral resources,^21^ self‐assessed competence in identifying IPV,^9^ lack of confidence in the ability to make referrals, discomfort in asking the IPV screening questions.^9^

Further research is needed to better understand the effective methods for screening IPV with MSFW patients. Observational and survey design studies helping to identify the factors influencing health‐care providers' decision to screen, as well as experimental studies testing which training mechanisms are most effective in increasing provider comfort and skill with IPV screening are needed. Additionally, community‐based focus group studies would help to expand on the unique strengths, challenges and cultural factors impacting health‐care practices caring for the MSFW population, as well as identify needed changes in policy, procedure and available training/resources to improve screening frequency, intervention and referral rates.

### Patient‐centered factors

Consistent with previous studies of Latina women experiencing IPV,^7^ participants indicated several culturally relevant factors that exacerbate IPV among the MSFW community including undocumented status,^7^ limited education,^38^ lack of English proficiency^7^ and changes from acculturation and economic demands.^24^, ^39^ Furthermore, participants also described barriers MSFW patients face to disclosing IPV, including having partners or other family members present, lack of transportation, language barriers, confidentiality/privacy concerns and gender of health‐care provider. While provider perception of patient‐centered factors is critical to understanding the issues they encounter in their workplace, mixed method studies comparing providers' and patients' perceptions from the same community will help to develop patient‐centered and informed programmes and protocols versus IPV screening practices strictly developed from the providers' positions of privilege. No matter how well intended, it is difficult to think from another person's social location so involving patients as research and protocol advisors is recommended for advancements in IPV screening in the MSFW community. Although previous researchers^40^ have documented the preferences of women in general regarding IPV screening (e.g., being screened in‐person, verbally and by female providers), no one has specifically considered the unique cultural and legal influences of screening and identification on the MSFW population.

### Clinic‐centered factors

Providers reported mixed responses pertaining to the support they receive from the clinics in which they serve. For instance, some participants reported limited access to behavioural health providers and/or interpreters. Without these resources, they hesitated screening. Therefore, program evaluation studies are needed to garner more empirical evidence on the merits of behavioural health provider and interpreter inclusion as members of the health‐care team serving the MSFW population. Future researchers should also seek to develop a screening tool for IPV that is empirically valid and reliable for use among MSFW patients and study its clinical, operational and financial impacts. Peek^41^ argued that without consideration of all three of these worlds of healthcare, attempts at transforming the health‐care system will fail.

### Community‐centered factors

Perhaps one of the most difficult challenges to overcome for MSFW women victimized by IPV is the lack of community resources available to them to lend aid. Some participants reported having community resources (e.g., women's shelters), but others reported having limited access or no access at all to such resources. Furthermore, participants acknowledged that because IPV is considered by many to be an accepted tradition within the MSFW community, few are willing to speak out against it. In order for these cultural norms to change, health‐care providers, policy writers and researchers each must do their part in their respective arenas to influence the change that the MSFW community needs. Initial steps towards doing this may include appointing representatives from the MSFW community to serve on committees, boards and task forces to ensure that their unique needs and ideas are addressed in any of these forums. Researchers are also encouraged to get involved in their communities and study the impact of federal, state, and local legislation, community service programming, and public health trends on groups not often represented but who are largely impacted.

## Limitations

There are two limitations to note based on the study design and sample. Because interviews were conducted via telephone, the depth of observation was limited to the participants' tone of voice, which may have influenced the manner in which data were interpreted. Furthermore, the use of the telephone may have created a barrier that impacted the flow of the interviews. For example, at various points, static noise would cause the conversation to cut out, creating an interruption in the conversation. However, the use of telephone also enhanced anonymity and provided flexibility of time, which may have led to more transparent disclosure of the participants' experiences and more participants being willing to participate. Ultimately, this method of interviewing was also utilized out of convenience to provide maximum accessibility to participants located across the United States.

Furthermore, all participants in this study were female health‐care providers and it is not known whether women versus men have differing perspectives on the MSFW female population with respect to IPV screening and treatment. Thus, future research studies should seek to attain the lived experiences of male health‐care providers, especially considering the unique gender‐related aspects of IPV.

## Conclusion

Based on the study's findings, health‐care providers would benefit from education and training on how to detect, interview and care for MSFW women experiencing IPV. Resources such as the MCN (www.migrantclinician.org) offer support and information about IPV among the MSFW population.

In summary, the phenomenon of screening for and addressing IPV with MSFW patients presented the participants with many opportunities for personal and professional reflection, growth and the opportunity to consider the ways in which the current health‐care practices in this area can improve. At the same time, it presented challenges that the participants continue to struggle through. Some of these challenges were self‐imposed, while others were imposed by the imperfect health‐care system in which the participants serve. Overall, it appeared that participants agreed that IPV among the MSFW community is a significant problem that needs to be better addressed by the health‐care system, but many participants were unsure how they could really make a difference. These participants were attempting to find the line between where their own responsibility as providers ends and the responsibility of the health‐care system at large to support its' providers begins.

A quick start strategy to promoting change would be with medical clinic administrators seeking or offering education and training to providers about existing state and local resources where they could send MSFW patients reporting IPV. In addition, culturally relevant education and training is needed in the community (e.g., performed in Latino churches, festivals, stores) where the patient‐to‐patient distribution of information can occur. Health‐care begins in the community where patients talk to one another so it makes sense for education to be a priority there too.

## Compliance with ethical standards

The authors declare that they have no conflicts of interest. This study was approved by a University and Medical Center Institutional Review Board (UMCIRB) and the Migrant Clinicians Network Institutional Review Board (MCN IRB). Confidentiality of participants was ensured using pseudonyms and identification numbers on raw data. All participants in this study completed an informed consent process approved by the aforementioned institutional review boards.

## Conflict of interest

No conflict of interests has been declared.
